# Substance Use and College Completion Among Two-Year and Four-Year College Students From a Nationally Representative Longitudinal Study

**DOI:** 10.7759/cureus.61297

**Published:** 2024-05-29

**Authors:** Janet E Rosenbaum

**Affiliations:** 1 Epidemiology and Biostatistics, State University of New York Downstate Health Sciences University, Brooklyn, USA

**Keywords:** adolescent substance abuse, young adult health, educational attainment, social determinants of health (sdoh), : adolescence

## Abstract

Young adults from disadvantaged populations access higher education through two-year colleges, but substance use research among young adults focuses on four-year colleges. Filling this research gap is important given recent policy changes that have increased marijuana availability for young adults. This study uses a subsample of college-enrolled participants from the National Longitudinal Study of Adolescent to Adult Health (Add Health) to evaluate whether substance use predicts educational attainment seven years later, comparing 888 students attending a two-year college with 1,398 matched students attending a four-year college. Matched students were identified using a propensity score method so that students were comparable on 15 measures, including precollege grades, precollege test scores, and precollege substance use. Compared with similar four-year college students, two-year college students were more likely to use methamphetamines, cocaine, or marijuana; more likely to report problematic substance use; and less likely to use alcohol. Two-year college students who used methamphetamines in the past year (incidence rate ratio (IRR) = 1.51, 95% CI (1.12, 2.04), p = 0.007) or past month (IRR = 1.69, 95% CI (1.09, 2.61), p = 0.02) or completed alcohol abuse treatment (IRR = 1.58, 95% CI (1.21, 2.07), p < 0.001) were less likely to complete college than two-year college students without those risk factors. Among the matched four-year college students, students who reported that drugs interfered with school or work in the past year (IRR = 1.84 (1.28, 2.64), p = 0.001), used cocaine in the past year (IRR = 1.47 (1.04, 2.08), p = 0.03), and used marijuana in the past year (IRR = 1.30 (1.07, 1.57), p = 0.007), past month (IRR = 1.31 (1.07, 1.61), p = 0.01), or ≥5 times in the past month (IRR = 1.44 (1.12, 1.85) p = 0.005) were less likely to complete college than the matched four-year college students without those risk factors. Substance use interventions should target both two-year and four-year college students. Two-year colleges that better accommodate students who complete substance use treatment may improve these students’ completion. Students who use marijuana or cocaine or whose drug use impairs functioning may benefit from an incremental approach of completing a two-year degree prior to transferring to a four-year degree rather than enrolling directly in a four-year program.

## Introduction

Degrees from both two-year and four-year colleges yield high economic and noneconomic returns [1], but college persistence is low and characterized by racial and socioeconomic disparities [2]. Excessive use of marijuana and alcohol is associated with lower completion of four-year college degrees [3], but little research has evaluated whether the same is true at two-year colleges [4].

Nonrepresentative studies have found that community college students are at least as likely to binge drink as four-year college students, adjusting for socioeconomic factors [5]. Most community colleges offer anti-alcohol education programs, and about half refer students with potential alcohol use disorders for treatment [6]. Some pilot studies have evaluated text-messaging anti-alcohol interventions among community college students [7].

Alcohol and illegal drug use are common during adolescence and emerging adulthood but often decline with age. Many young adults (ages 18-25) who meet the criteria for problem drinking decrease alcohol consumption over seven years [8]. Continued substance and alcohol use can be a marker for difficulties in the transition to adulthood, including the completion of schooling. The existing longitudinal research on whether substance use predicts long-term educational attainment focuses on four-year college completion. For instance, the Coronary Artery Risk Development in Young Adults study, a random sample from four cities, found that past-month marijuana users and heavy drinkers were less likely to have graduated college within 10 years [9] and that high levels of alcohol consumption at ages 18-30 predicted lower chances of college graduation and lower occupational prestige 15 years later [10]. Marijuana is associated with lower grade point averages (GPAs) among four-year college students [3,11].

As a result of community colleges’ lower entry barriers, community college students are highly similar to high school graduates who do not matriculate in postsecondary education, with just slightly higher precollege socioeconomic status, grades, and test scores [12]. By contrast, there is substantial selection bias in four-year colleges compared with the alternatives, such as a two-year college or not attending a postsecondary school [13]. Prior to entering college, four-year college students have vastly higher grades and test scores, socioeconomic status, better health, and fewer health risk behaviors than adolescents who did not matriculate in four-year colleges [12].

Disadvantaged youth primarily access higher education through community colleges, which some regard as a promising vehicle for social mobility [14]. Community colleges are affordable and have reduced traditional barriers to admission for disadvantaged youth who would be otherwise unlikely to attend college [15]. Community college was formerly a negligible portion of the postsecondary educational system, but currently, community college students comprise 46% of undergraduates (in the most recent available data from 2016; Table 304.80) [16]. Community colleges have low entry barriers, including open admissions, schedules that accommodate working students, including night and weekend classes, convenient locations, low tuition, and free tuition in some states (e.g., Tennessee). Completion, rather than access, remains the chief challenge for community college students. Among community college students, about 15% finish an associate’s degree or certificate within three years, and 45% leave school with no credential [17]. Half of former community college students cite “personal reasons” as their explanation for leaving school, and about twice as many cite “family” or "finances,” which could include many risk factors not measured by United States national education surveys, such as current and past drug use [2].

Recent changes to the marijuana policy environment have increased the availability of marijuana to college students. Evidence from long-term longitudinal studies in the current cohort of college students is needed to show the associations between marijuana and educational attainment, the extent to which marijuana will substitute for the use of other drugs, and how illegal drug use will affect educational attainment. This study supplements the literature on community college completion by using a nationally representative health dataset from a prior cohort of college students to identify associations between drug and alcohol use, matriculation in a two- or four-year college, and educational attainment. We evaluate whether students attending two-year and four-year colleges who also use alcohol, marijuana, and illegal drugs are less likely to have attained any postsecondary degree seven years later.

## Materials and methods

To minimize confounding and avoid reverse causality, we use propensity matching methods on precollege variables to identify similar students attending two-year and four-year colleges. This matching is particularly important because substance use is associated with selection into a two-year versus four-year college [18].

Participants

These hypotheses were tested in the National Longitudinal Study of Adolescent to Adult Health (Add Health), a nationally representative sample of US students who were in grades 7-12 in 1995. This study uses the 1995 (baseline), 2001, and 2008 waves [19]. The 1995 baseline survey was used instead of the 1996 wave 2 survey because the sample size was larger; a subset of baseline respondents was not invited to participate in wave 2, although they were asked to participate in waves 3 and 4 as described elsewhere [19].

We measured precollege variables from the wave 1 in-home interview in 1995, when the sample was ages 12-18; college enrollment in the wave 3 in-home interview in 2001, when the sample was ages 18-24; and graduation status in the wave 4 in-home interview in 2008, when the sample was 25-31. By analyzing a seven-year span, these data can identify completion, even when degrees take more than the usual time for completion, such as BAs taking over four years. The sample comprises 4,218 high school graduates without postsecondary credentials enrolled in a four-year or community college in 2001. High school equivalence credentials (GEDs) were not counted as high school diplomas because of observed differences in their employment and health outcomes in other studies [20].

Measures

Control Variables

We matched on control variables that were precollege variables associated with both substance use during college and college completion: socioeconomic status, demographics, education, substance use, and having ever been pregnant during high school. The background precollege measures were measured in the baseline in-home interview in 1995, when the respondents were ages 12-18. The socioeconomic status variables were parent-reported education level, parent-reported household income (on a log scale, singly imputed with an indicator for missingness), and parent-reported response to whether the household has enough money to pay bills. Demographics were measured by whether the respondent and/or parent were born in the US, race, ethnicity, and age. Baseline substance use was a number of friends who smoked cigarettes, lifetime use of alcohol while unsupervised, and lifetime use of marijuana. Marijuana use during high school has been associated with college dropout [18], but this study matches drug use during high school. Educational variables were whether the respondent intends to go to college, standardized test (percentile on the Peabody Vocabulary Test), school attachment problems, history of school suspension or expulsion, and self-reported GPA on a 4.0 scale in English, history, science, and math.

Exposure Variables: Substance Use

Substance use was measured in 2001 at ages 18-24 and included five quantitative variables about alcohol use, one quantitative variable about marijuana use, and 11 binary variables about illegal drug use and alcohol abuse treatment. The number of episodes of alcohol use in the past year was the response to the question “During the past 12 months, how many days did you drink alcohol?” The number of episodes of binge drinking in the past year was defined as the number of times in the past year that the respondent consumed “5 or more drinks in a row.” The number of alcoholic drinks typically consumed in one episode of drinking was the response to the question “Think of all the times you have had a drink during the past 12 months. How many drinks did you usually have each time? A ‘drink’ is a glass of wine, a can of beer, a wine cooler, a shot glass of liquor, or a mixed drink.” The number of episodes of binge drinking in the past two weeks was defined as “five or more drinks on a single occasion, for example, in the same evening” for males and “four or more drinks on a single occasion, for example, in the same evening” for non-males. The number of times drunk was the response to the question “During the past 12 months, on how many days have you been drunk or very high on alcohol?” The number of episodes of marijuana use in the past month was the response to the question “During the past 30 days, how many times have you used marijuana?”

The binary variables were self-reported marijuana use in the past month and past year, cocaine use in the past month and past year, methamphetamine use in the past month and past year, injection drugs in the past month and past year (fewer than 10 cases), “other illegal drugs” in the past month and past year, whether drugs interfered with life in the past year, and obtaining treatment for alcohol abuse. “Other” illegal drugs were not listed in the survey, but they could include any illegal drug other than cocaine and marijuana, such as MDMA/ecstasy or LSD.

College Enrollment

College enrollment in two-year versus four-year colleges was measured in 2001 at ages 18-24 as the response to the question “Is this a high school, a two-year college, a four-year college, or a graduate school?”

Outcome Variable: Educational Attainment

Educational attainment was measured in 2008 at ages 25-31 as the highest degree listed in a detailed history of every degree attained and the date: certificate, associate’s degree (AA), or bachelor’s degree or above (BA+). Respondents attained their highest degree three to 13 years after baseline.

Statistical analysis

Bivariate Analysis

We compared substance use by two-year and four-year college students using the Mann-Whitney test for dichotomous substance use measures and the Kruskal-Wallis test for non-dichotomous categorical substance use measures.

Multivariate Analysis 

We evaluated whether each substance use condition was more common among community college students than among four-year students using a multivariable Poisson regression model with robust standard errors estimated with the sandwich estimator: first in the raw data and then after the propensity score matching model to demonstrate that matching does not change the direction of association. Poisson regression with robust standard errors yields consistent and unbiased estimators that are also easily interpretable [21] because these regressions yield incidence rate ratios (IRRs) that can be interpreted as relative risks; they are not odds ratios.

The control variables in the regression model were demographics (male gender, age, Hispanic, Asian, and African American self-identities); deviance (ever used marijuana, number of friends who smoke, having ever had an out-of-school suspension, and ever pregnant); and socioeconomic status (parent-reported household income, test score, GPA, GPA missing, school attachment, and college expectancies).

Propensity Matching Method

Differences in substance use between two-year and four-year college students could be due to their college setting, but they could also be due to adolescents with specific precollege drug habits choosing two-year or four-year colleges. This paper matched baseline factors that may be important in students’ self-selection into two-year versus four-year colleges, measured in 2001. We implemented 1:1 exact and nearest-neighbor Mahalanobis matching within propensity score calipers using the MatchIt library in the R statistical package [22].

All factors used for matching were measured at baseline (1995), except age, which is computed at wave 3 (2001) from the self-reported birth date and the date of the interview. We refined the matching model until there were no significant differences between the two groups of college students on key variables. For each two-year college student, we identified all four-year college students who matched exactly on three binary variables: African American or Black self-identification, grades being unreported, and a binary measure of college expectancies. After exact matching on these three binary variables, we limited the pool of potential four-year college student matches to participants whose estimated propensity scores were within 0.25 standard deviations. Within the pool of potential four-year college students, we chose the one four-year college student that is closest according to the Mahalanobis matching; we matched with replacement, meaning that each four-year college student may be used more than once, which improves post-matching balance. The Mahalanobis metric estimated the distance between participants based on age, GPA, parent-reported household income, and standardized test score. The propensity score model used for estimating the propensity score calipers included baseline (precollege) measures for self-identified male gender (versus genders other than male), having ever been suspended, having ever been pregnant, having ever used marijuana, having ever used alcohol unsupervised, mother having a college degree, number of friends who smoke, and an aggregate measure of school attachment problems.

Post-matching Multivariate Analysis

After matching, we repeated the multivariable Poisson regression with robust standard errors, controlling for demographics (male gender, age, Hispanic, Asian, and African American self-identities); deviance (ever used marijuana, number of friends who smoke, having ever had an out-of-school suspension, and ever pregnant); and socioeconomic status (parent-reported household income, test score, GPA, GPA missing, school attachment, and college expectancies). As in the pre-matching multivariate analysis, the Poisson yielded estimates of IRRs, which can be interpreted as relative risks; they are not odds ratios.

Ethical considerations

This paper was previously presented as a poster at the 2011 American Public Health Association annual meeting and posted on the Research Square preprint server on October 3, 2019. This study was ruled not human subjects research by the institutional review board of the State University of New York Downstate Health Sciences University (FWA number 00003624; study number 440410-1).

## Results

Substance use by two-year versus four-year college students

Community college students were more likely than four-year college students to have used methamphetamines (past year and past month), cocaine (past year), injection drugs (past year and past month), and marijuana more times in the past month, according to bivariate analysis (Table 1). Four-year college students reported more frequent alcohol use, binges, and episodes of intoxication in the past year and more alcohol binges in the past two weeks, but community college students were more likely to have been in alcohol abuse treatment and reported drinking more alcoholic drinks per night.

**Table 1:** Prevalence of substance use by two- and four-year college students

Substance use and associated behaviors	Four-year college students (n = 2,813)	Two-year college students (n = 1,398)	p
Marijuana use in the past year (%)	32.2	34.3	0.17
Cocaine use in the past year (%)	4.91	6.83	0.01
Cocaine use in the past month (%)	1.03	1.49	0.19
Methamphetamines in the past year (%)	1.1	3.35	<0.001
Methamphetamines in the past month (%)	0.28	1.14	<0.001
Other illegal drugs in the past year (%)	9.81	10.8	0.34
Other illegal drugs in the past month (%)	2.1	2.21	0.82
Injection drugs in the past year (%)	0.21	0.57	0.06
Injection drugs in the past month (%)	0	0.36	<0.001
Drugs interfered with work or school in the past year (%)	3.23	3.91	0.26
Alcohol abuse treatment in the past year (%)	2.6	3.91	0.02
Number of episodes of marijuana use in the past month (mean (SD))	2.17 (7.83)	2.73 (8.69)	0.04
Number of episodes of consuming more than five alcoholic drinks in the past year (mean (SD))	20.5 (45.7)	15.6 (39.6)	<0.001
Number of episodes of inebriation in the past year (mean (SD))	18.3 (38.7)	13.2 (34.5)	<0.001
Number of episodes of alcohol use in the past year (mean (SD))	45.1 (64.5)	34.5 (56.8)	<0.001
Number of episodes of more than five alcoholic drinks in the past two weeks (mean (SD))	1.03 (2.02)	0.78 (1.68)	<0.001
Usual number of alcoholic drinks in a drinking episode (mean (SD))	4.13 (2.59)	4.35 (2.98)	0.15

**Figure 1:**
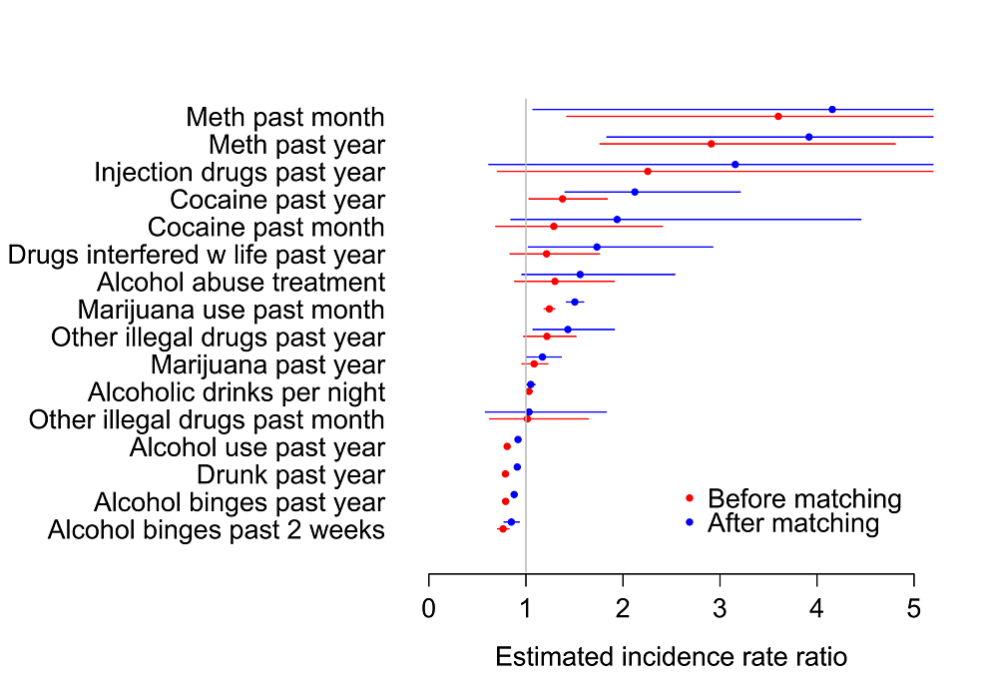
IRR of substance use among two-year college students versus four-year college students in the full sample (n = 4,218) and matched sample (n = 2,286) A ratio greater than 1 means that the frequency is higher among community college students than among four-year college students. IRRs were estimated by a Poisson working model in full and matched samples. Multivariate results are shown in red for the raw data and in blue for the matched sample. Control variables were male gender, age, race/ethnicity (Hispanic, Asian, or African American), 1995 marijuana use, friends’ smoking, out-of-school suspension history, parent income, test score, GPA, GPA missing, school attachment, ever pregnant, and college expectancies. GPA, grade point average; IRR, incidence rate ratio

Community college students were more likely to have used methamphetamines (past year and past month), cocaine (past year), and marijuana in the past month in multivariate analysis before matching (Figure 1). Four-year college students were more likely to have used alcohol in the past year, to have been drunk in the past year, and to have had more alcohol binges in both the past year and the past two weeks.

**Figure 2:**
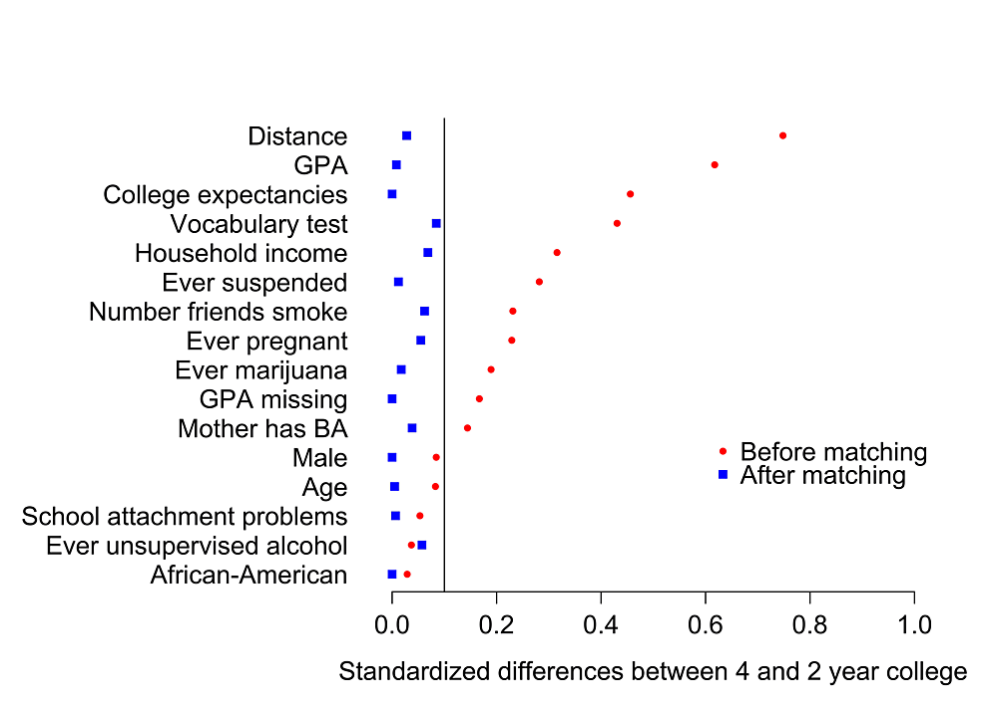
Precollege characteristics of two-year college students and four-year college students, before and after matching The absolute values of standardized differences are plotted; standardized differences <0.1 are not significant.

Matching identified 888 four-year college students (out of 2,813 four-year college students) that were most similar to the 1,398 two-year college students. Before matching, community and four-year college students differ substantially on several baseline factors: the estimated propensity of attending two-year versus four-year college (computed with the propensity model variables listed above), GPA, whether they expect to go to college, vocabulary test score, household income, having ever been suspended, having friends who smoke, having ever been pregnant, having used marijuana, not reporting grades on the wave 1 survey, and mother having a college degree (Figure 2). After matching, these baseline variables were no longer significantly different.

**Figure 3:**
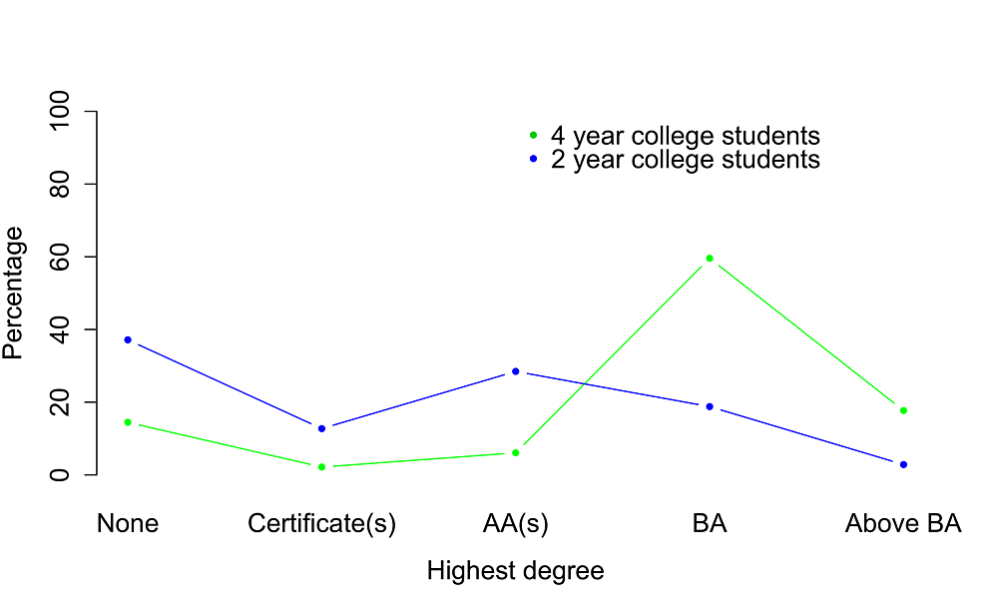
Educational attainment for two-year college students (n = 1,398) and four-year college students (n = 2,813)

After matching on baseline factors that may have otherwise confounded the relationship between drug use and college choice, two-year college students were more likely to have used methamphetamines (past month and past year), cocaine (past year), marijuana (in the past year and past month), and other illegal drugs in the past year, to say drugs interfered with their lives in the past year, and to drink more alcoholic beverages per drinking episode than students at four-year college (Figure 3). Matching reduced most associations between drug use and choice of two-year versus four-year college toward the null of no association (Figure 1).

Associations between substance use and educational attainment for two-year versus four-year college students

Most (over 60%) of the four-year college students in this sample attained a bachelor’s degree, and small numbers attained certificates or associate degrees, but almost 20% attained no postsecondary credential (Figure 3). Almost 40% of the two-year college students in this sample attained no postsecondary credential.

**Figure 4:**
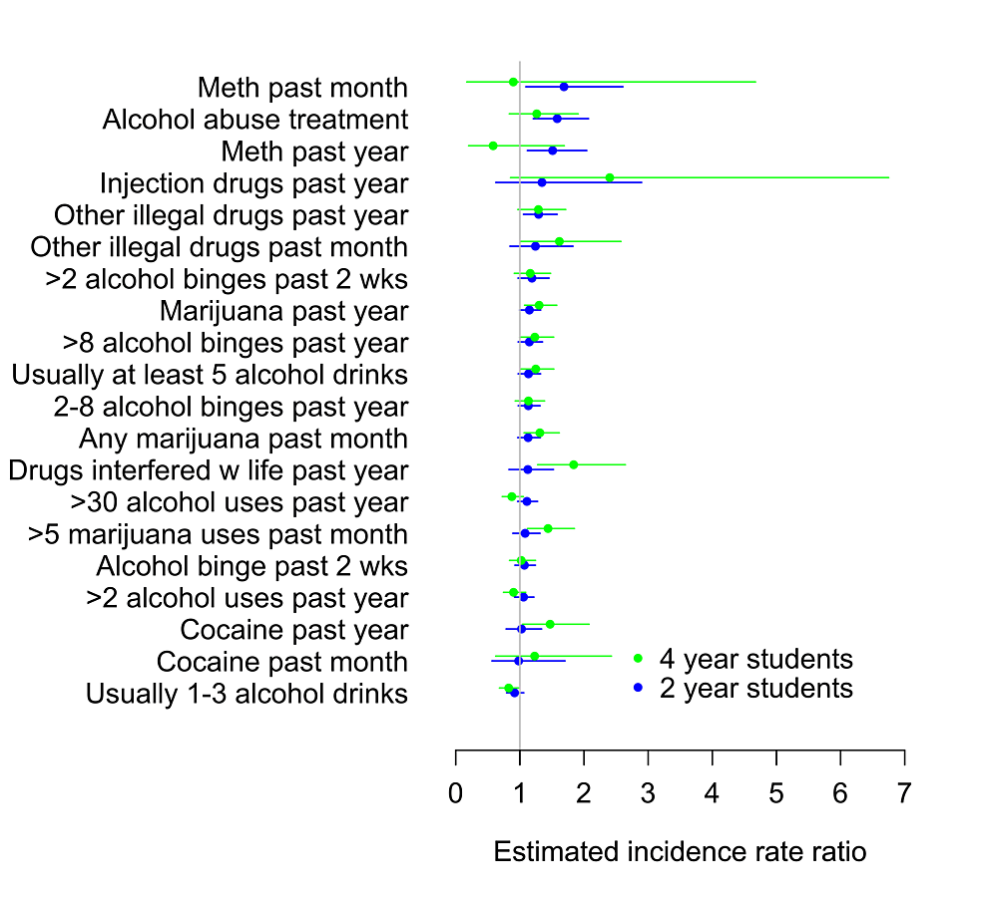
Relative risk of college non-completion among two-year college students (blue, n = 1,398) and four-year college students (green, n = 888) in the matched sample

Two-year college students with the following substance use factors were less likely to have attained any postsecondary degree within seven years of reporting being enrolled compared with students without those substance use factors: having been in alcohol abuse treatment (IRR = 1.58, 95% CI (1.21, 2.07), p < 0.001), having used methamphetamines in the past year (IRR = 1.51, 95% CI (1.12, 2.04), p = 0.007) or past month (IRR = 1.69, 95% CI (1.09, 2.61), p = 0.02), or having used other illegal drugs in the past year (IRR = 1.29, 95% CI (1.06, 1.58), p = 0.01) (Figure 4).

Four-year college students with the following substance use factors were less likely to have attained any postsecondary degree within seven years of reporting being enrolled, compared with students without those substance use factors: reporting that drugs had interfered with school or work (IRR = 1.84 (1.28, 2.64), p = 0.001); having used cocaine in the past year (IRR = 1.47 (1.04, 2.08), p = 0.03); injection drugs in the past year (IRR = 2.40 (0.85, 6.75), p = 0.097); having used marijuana in the past year (IRR = 1.30 (1.07, 1.57), p = 0.007), past month (IRR = 1.31 (1.07, 1.61), p = 0.01), or at least five times in the past month (IRR = 1.44 (1.12, 1.85), p = 0.005); having “binged” on alcohol more than eight times in the past year (IRR = 1.23 (0.996, 1.53), p = 0.05); reporting that usually they consume at least five alcoholic drinks at a time (IRR = 1.25 (1.02, 1.53), p = 0.03); or having used “other” illegal drugs in the past month (IRR = 1.62 (1.01, 2.58), p = 0.04) (Figure 4). Four-year college students who reported that they usually drink one to three alcoholic drinks were more likely to attain a postsecondary degree than students who reported that they usually do not drink alcohol (IRR 0.83, 95% CI (0.68, 1.00), p = 0.05).

## Discussion

With recent public policy changes, marijuana is increasingly available to college students [23]. The results in this cohort of college students find that students at four-year colleges who use marijuana at various intensities are less likely to have attained a postsecondary degree within seven years, but the same is not true for students at two-year colleges. The use of a variety of other drugs predicts a lower likelihood of completion.

Although less studied than four-year college students, two-year college students engage in more drug use risk behaviors than matched four-year college students, with greater use of methamphetamines, cocaine, and marijuana and greater life impairment from substance use but a lower likelihood of binge-drinking alcohol, even after matching on precollege substance use, test scores, grades, socioeconomic, and demographic factors that may have affected students’ self-selection into two-year versus four-year colleges. Substance use appears to be more associated with four-year college non-completion than two-year college non-completion, even after using matched sampling to adjust for observed factors that predict selection into two-year versus four-year college. Marijuana use predicts a lower likelihood of graduation from four-year college but not from two-year college. The increased availability of marijuana and changed social norms due to decriminalization or legalization make this disparity especially germane [23]. Increased marijuana use may magnify racial and socioeconomic disparities in college completion among four-year college students.

Despite serving students from more socioeconomically disadvantaged backgrounds who may have greater health needs and lower healthcare access, two-year colleges generally do not have as many resources or services to help students with substance use [6].

Past research in Add Health finds that high school students diagnosed with depression are more likely to select a two-year college than a four-year college [24]. The greater substance use among two-year college students could be partially explained by some two-year college students with depression self-medicating with substance use.

The finding that alcohol abuse treatment predicts a greater risk of non-completion for two-year college students is consistent with past research finding that non-childbirth hospital admissions also predict a greater risk of non-completion [24]. Two-year colleges need to better accommodate students’ health needs and help students resume college after medical treatment.

Students who attend four-year colleges engage in more alcohol use, especially binge drinking. Four-year college students may have adopted these alcohol use patterns at college. A longitudinal study of beginning college students at the University of Texas found social motives (agreeing that, e.g., attending social events is important) predict greater alcohol use and that academic motives are protective against alcohol use for females during the second year of college [25]; beginning college students’ alcohol use is associated with lower class attendance [26], which could lead to non-persistence if repeated.

Moderate alcohol use may be protective for four-year college students’ college graduation relative to no use because, in some cases, moderate alcohol use may signal greater social integration at college, consistent with Tinto’s theory that students who become well integrated, both academically and socially, are more likely to persist in college than students who are not well integrated [27]. Alcohol use is central to the social life at many residential four-year colleges, and students who are able to navigate these social spheres while engaging in moderate alcohol use may have greater social integration at these colleges and thus maintain the social connections needed to graduate college. This evidence is consistent with past research finding that moderate alcohol use predicts higher wages, including a study using the Add Health data that explains the association between moderate alcohol use and wages by sociability [28] and the results of social network analysis in Add Health that alcohol use is associated with greater popularity [29]. Alternatively, moderate alcohol use may signal resilience or greater executive function, which also increases the likelihood of four-year college graduation.

Students with greater levels of illegal drug use or past alcohol abuse may be more likely to enroll in two-year college, or they may have initiated greater levels of illegal drug use after beginning two-year college. Students who began to use substances at greater levels after our precollege variables were measured may have chosen to enroll in a two-year college, perhaps after a four-year college. Regardless of the reasons for the higher substance use among two-year college students, two-year colleges need greater resources to help their students.

Strengths and limitations

This study uses the Add Health dataset to study two-year college students because information about substance use is not available in standard Department of Education datasets, such as the Beginning Postsecondary Students Longitudinal Study (BPS) data. The sample is more diverse than previous studies of risk behaviors among two-year college students because it is derived from a nationally representative sample of students enrolled in middle and high school. The estimates of the proportion of two-year college students obtaining no degree are comparable to estimates from other national data, including BPS and the Educational Longitudinal Study [30].

Matched sampling identified students from two-year and four-year colleges who were comparable on baseline variables including precollege substance use, grades and test scores, and socioeconomic status. Matching reduces the likelihood that differences between two-year and four-year college students were due to selection into two-year college by students with greater substance use earlier in adolescence. The matching model was balanced on 15 measures of precollege educational background and socioeconomic status that are known to be highly associated with educational attainment, but it is nonetheless possible that some unmeasured confounding remained after matching and regression analysis that may have reduced the average educational attainment of two-year college students relative to four-year college students.

The sample comprised youth enrolled in two-year and four-year colleges in 2001, so these findings may not apply to the current cohort of students enrolled in two-year and four-year colleges. These delays are a necessary disadvantage for obtaining long-term outcomes. In this case, graduation/educational attainment was measured seven years after the measurement of college enrollment, longer than the BPS datasets that follow up at five years for the 2012 cohort and six years for the 2003 cohort.

Due to the data being self-reported, it is possible that the substance use measures were misreported. If data had been collected retrospectively, we might be concerned about recall bias - that participants with lower educational attainment would differentially misreport substance use. However, the data were collected prospectively with educational attainment measured seven years after substance use, so it is unlikely that the underreport would have been differential by educational attainment.

This study uses an observational design because it is not possible to randomly assign people to two-year or four-year colleges. The proposed federal policies for “free community colleges” that would improve students’ access to higher education could facilitate quasi-experimental research designs to attempt to replicate these findings.

## Conclusions

Two-year college students engage in a wider variety of substance use behaviors than comparable four-year college students and require appropriate substance use education or interventions. These results suggest that substance use education and interventions for two-year college students should include substances beyond alcohol, including methamphetamines and cocaine. This research suggests that students who have received alcohol treatment may need their college’s help to return to classes and integrate socially and academically after the treatment.

Substance use is germane to college choice and college completion, which is a key social determinant of health. The changing marijuana policy environment has increased marijuana availability, including among college students, making it particularly important to collect data about substance use in the postsecondary educational datasets used by policymakers, two- and four-year colleges, and public health officials. All college students would benefit from substance use education about the cardiovascular and other less-publicized health effects of marijuana.
